# Clinical significance of TM4SF1 as a tumor suppressor gene in gastric cancer

**DOI:** 10.1002/cam4.1494

**Published:** 2018-04-17

**Authors:** Xing‐Chun Peng, Zhi Zeng, Yu‐Ning Huang, Yun‐Chao Deng, Guo‐Hui Fu

**Affiliations:** ^1^ Pathology Center Shanghai General Hospital/Faculty of Basic Medicine Shanghai Jiao Tong University School of Medicine Number 280, South Chong‐Qing Road Shanghai 200025 China; ^2^ School of Basic Medical Sciences Hubei University of Medicine Shiyan 442000 Hubei Province China; ^3^ Department of Pathology Renmin Hospital of Wuhan University No.99, Ziyang Road Wuchang District, Wuhan 430060 Hubei Province China; ^4^ Department of Gastroenterology Renmin Hospital of Wuhan University No.99, Ziyang Road Wuchang District, Wuhan 430060 Hubei Province China

**Keywords:** Biomarker, gastric cancer, prognosis, TM4SF1

## Abstract

Transmembrane‐4‐L‐six‐family member‐1 (TM4SF1), a tumor‐associated antigen, is overexpressed in most epithelial cell carcinomas and a potential target for antibody‐mediated therapy. However, the role of TM4SF1 in gastric cancer has not been elucidated. The aim of this study was to investigate the clinical significance of TM4SF1 expression in gastric carcinoma (GC) tissues using 152 GC tissue samples and matched adjacent nontumor tissue samples analyzed by immunohistochemistry, and 13 fresh GC tissue samples analyzed by Western blotting. The results showed that TM4SF1 was heterogeneously expressed in normal gastric mucosa, with a high expression rate in fundus mucosa. Higher levels and strong expression rate of TM4SF1 were associated with GC tissues of higher‐grade differentiation. TM4SF1 levels were lower in gastric cancer tissues than gastric noncancerous tissues. Expression of TM4SF1 was not correlated with USP10 (*P *=* *0.157), S100A12 (*P *=* *0.479), p53 (*P *=* *0.249), or Ki67 (*P *=* *0.166) in GC. The expression of TM4SF1 was significantly and negatively correlated with depth of invasion (*P *=* *0.031), nodal metastasis (*P *=* *0.042), TNM stage (*P *=* *0.030), and Lauren classification (*P *=* *0.026). There was no significant correlation between TM4SF1 expression and age, gender, tumor size, or distant metastasis (*P > *0.05). The expression of TM4SF1 was associated with well overall survival (*P *=* *0.0164). The 5‐year survival rate for patients with GC showing TM4SF1 positive was 58.82% (10/17), and the median survival time was 78 months, higher than that (12.90%, 12/93) of patients who were TM4SF1 negative, whose median survival time was 62 months. These data suggested that low expression of TM4SF1 is associated with carcinogenesis and development, tumor progression and invasion of gastric cancer, and poor overall survival of patients with GC. TM4SF1 is a tumor suppressor for GC and a novel prognostic marker for patients with GC.

## Introduction

Gastric cancer (GC) is the most common malignant gastrointestinal cancer and the second leading cause of cancer‐related death worldwide 1. The prognosis of GC remains poor despite of advances in the therapeutic means. Many patients are found to carry tumors in an advanced stage at the time of diagnosis, which are unresectable. The overall survival (OS) of these patients was less than 12 months 2. It remains of great clinical importance to identify new biomarkers for early diagnosis, targeted treatment, and prognostic evaluation in gastric cancer.

Transmembrane‐4‐L‐six‐family member‐1 (TMSF1), also known as tumor‐associated antigen L6, is a 22 kDa four‐transmembrane‐domain protein 3, 4, 5, 6. TM4SF1 is abundantly expressed on the plasma membrane and intracellular vesicles in various human epithelial malignancies including lung, breast, colon, ovarian, renal, and prostate carcinomas, and low expressed in normal vascular endothelium 7, 8. Immunofluorescence microscopy also demonstrated that TM4SF1 deposits in the cytoplasm, and tentatively in nuclei of both cultured endothelial cells and tumor cells 9. TM4SF1 has been shown to be associated with growth, motility, invasion, and metastasis of tumor cells 8, 10. Overexpression of TM4SF1 has now been strongly linked to poor prognosis of muscle‐invasive bladder cancer 11, pancreatic cancer 12, 13, glioma 14. Loss of TM4SF1 contributes to the invasion and migration of pancreatic cancer cells 15. TM4SF1 has drawn much attention to develop targeted monoclonal antibody‐based cancer therapy 16, 17. However, the exact role of TM4SF1 in cancer including human gastric cancer development and progression remains unclear 18.

In this study, we investigated clinical significance of TM4SF1 in gastric cancer by analyzing TM4SF1 expression in GC and noncancerous tissue samples and their correlation with and clinicopathological factors, the expression of USP10, S100A12, p53 and Ki67, and overall survivals. Our data indicated TM4SF1 as a tumor suppressor gene was associated with carcinogenesis and development, tumor progression and invasion of gastric cancer, and poor overall survival of patients with GC and was an independent predictor for prognosis of patients with GC.

## Materials and Methods

### Patients and specimens

A total of 152 patients with GC (101 men and 51 women; age range, 31~84 years; median age 58 years) hospitalized in the Renmin Hospital of Wuhan University between January 2009 and June 2013 were included in this study. None of these patients had received radiotherapy or chemotherapy prior to surgery. Demographic and clinical pathological data, including age, sex, tumor size, tumor differentiation, Lauren classification, and TNM stage were collected in the hospital using a standard interviewer‐administered questionnaire and/or medical records. All tumors were staged according to the International Union Against Cancer (UICC) Tumor‐Node‐Metastasis (TNM) staging system (7th edition). A total of 152 GC tumor tissue samples from these patients with GC and matched adjacent normal gastric mucosa tissue samples were formalin‐fixed and paraffin‐embedded. Among these patients, a total of 110 patients who received the same surgical therapy excluding preoperative chemotherapy or radiotherapy were followed up until July 31, 2015. The survival status was confirmed by clinic records and patient or family contact. The duration from the date of surgical treatment to the date of death was defined as the overall survival. USP10 and S100A12 were stained using anti‐USP10 (1:300; Abcam, Cambridge, MA) and anti‐S100A12 (1:250, Sigma‐Aldrich, St. Louis, MO), respectively. Ki67 and p53 were stained using anti‐Ki67 (1:100, Abcam) and anti‐p53 (1:200, Santa Cruz Biotechnology, California), respectively. The degree of p53 and Ki67 expression as stained in the nuclei was defined and scored as the following: 0, <10% nuclei were positively stained; 1 + , 10~25%; 2 + , 26~50%; 3 + , >50%. USP10 and S100A12 expression was scored according to a four‐tier grading system (scores: 0 = absent, 1 = weak, 2 = moderate and 3 = strong staining). Score ≤1 was considered negative, and score >1 was considered positive. The data on USP10, S100A12, P53, and Ki67 staining were provided by the department of pathology in the Renmin Hospital of Wuhan University. Additional 13 paired samples of GC and noncancerous gastric mucosa were gathered, snap‐frozen immediately in liquid nitrogen, and stored at −80°C following surgery for Western blot analysis**.** The research was approved by the Ethics Committee of Renmin Hospital of Wuhan University. The written informed consent was obtained from each patient involved in the study.

### Immunohistochemistry and quantification of TM4SF1 staining

Paraffin‐embedded sections (4 *μ*m thick) were deparaffinized, rehydrated, and heated for 10 min in 10 mmol/L of sodium citrate (pH 6.0) to retrieve antigen. Endogenous peroxidase was quenched with 3% H_2_O_2_. After incubation with primary antibody (anti‐TM4SF1, 1:200, Abcam) at 4°C, the sections were incubated with horseradish peroxidase‐labeled polymer conjugated with secondary antibody (Max Vision^™^ Kits) at room temperature for 15 min, followed by development in diaminobenzidine and lightly counterstained with hematoxylin. In the negative controls, all procedures were the same except no primary antibody was added. The immunohistochemical results were independently assessed by two pathologists who were blinded to the clinical data. Five random fields of view at ×200 were examined for each section. The degree of TM4SF1 expression in the tumor tissues was measured by a grading system: 0, negative staining; 1, light yellow; 2, light brown; 3, dark brown. Score 0 was defined as TM4SF1 negative, and Scores 1–3 were TM4SF1 positive.

### Western blotting

The homogenized gastric cancer and noncancer samples were lysed in RIPA lysis buffer. The lysate was centrifuged (13800 g) at 4°C for 30 min. The supernatant was used for Western blotting analysis. The aliquots containing approximate 20 *μ*g protein each were denatured with sodium dodecyl sulfate (SDS) loading buffer, resolved by electrophoresis in a 10% SDS polyacrylamide gel, and then transferred onto a polyvinylidene fluoride membrane. After blocking with 5% nonfat milk for 1 h, the membranes were incubated with a rabbit monoclonal antibody against TM4SF1 (1:1000 dilution, Abcam) or mouse anti‐human GAPDH monoclonal antibody (1:2000 dilution, Sigma‐Aldrich) at 4°C overnight. After washing with Tris‐buffered saline with Tween‐20 (TBST) for three times with 10 min each, the membranes were probed with the horseradish perioxidase (HRP)‐conjugated mouse anti‐rabbit IgG antibody (1:2000 dilution, Sigma Chemical Co., St Louis, MO) at room temperature for 1 h. After washing with TBST, the membranes were developed using a chemiluminescence phototope‐horseradish peroxidase kit according to the manufacturer's instructions (Pierce, Rockford, IL). The band intensity was measured by densitometry using Quantity One software (Bio‐Rad, Hercules, CA). The TM4SF1 protein level was normalized to that of GAPDH.

### Statistical analysis

Statistical analysis was analyzed with SPSS 13.0 statistical software (SPSS, Inc., Chicago, IL). The Students's *t*‐test and one‐way analysis of variance test were used to compare data between different groups. The chi‐square and Fisher's exact tests were used to analyze the statistical significance of the relationship between TM4SF1 expression and the clinicopathological factors of GC. The correlation between TM4SF1 expression and USP10, S100A12, Ki67, p53 expression was analyzed by Spearman's rank correlation analysis. Survival curves were drawn according to the Kaplan–Meier method, and the log‐rank test was applied to compare the survival curves. *P < *0.05 was considered significant. *r* was defined as the strength of correlation between TM4SF1 and USP10, S100A12, P53. |*r*|>0.5 was considered strong correlation, and |*r*|<0.5 was considered weak correlation.

## Results

### Expression and location of TM4SF1 in normal gastric mucosa

**Figure 1:**
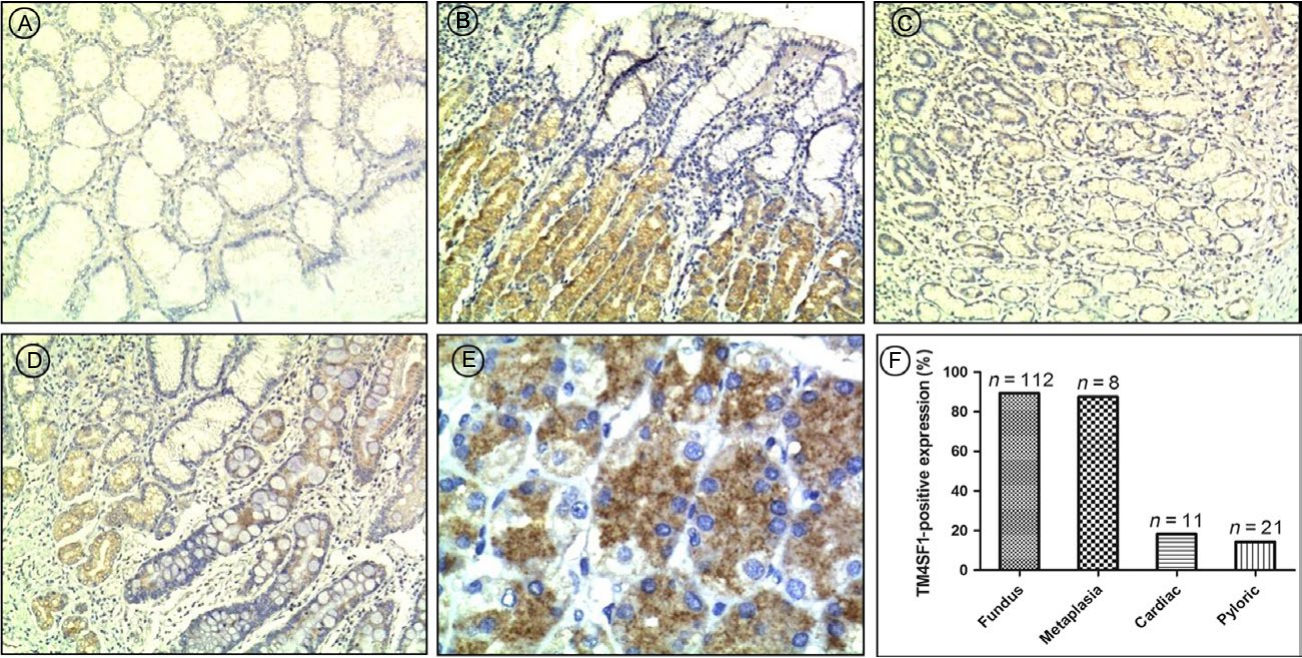
Expression and location of TM4SF1 in normal gastric mucosa. TM4SF1 expression in normal gastric mucosa was determined by immunohistochemtry. (A) cardiac mucosa, (B) fundus mucosa, (C) pyloric mucosa, (D) intestinal metaplasia mucosa. (E) The TM4SF1 staining in the chief cells of fundus glands and in the parietal cells. (F) Gastric tissue‐specific expression rates of TM4SF1.

To investigate expression and location of TM4SF1 in normal gastric mucosa, we performed immunohistochemical staining using paraffin sections of normal gastric mucosa samples. The results showed that there was no TM4SF1 staining in most cardiac and pyloric mucosa (Fig. 1A,C). TM4SF1 staining was seen in fundus mucosa (Fig. 1B). Notably, TM4SF1 protein is mainly expressed in the inherent glands, but not in the cervical mucus cells in the fundus mucosa. TM4SF1 staining was also seen in intestinal metaplasia mucosa (Fig. 1D). The TM4SF1 staining was more intense in the chief cells of fundus glands than that in the parietal cells (Fig. 1E). Statistical analysis indicated that TM4SF1 expression rate was higher in fundus mucosa tissues (89.29%, 100/112) than those of cardiac (18.18%, 2/11) and pyloric (14.29%, 3/21) mucosa tissues (Fig. 1F). There was no significant difference in TM4SF1‐positive expression rates between fundic and metaplasia mucosa (87.50%, 7/8) (Fig. 1F). These data suggested that TM4SF1 was heterogeneously expressed in normal gastric mucosa, with a high expression rate in fundus mucosa.

### Higher levels and strong expression rate of TM4SF1 were associated with GC tissues of higher‐grade differentiation

**Figure 2:**
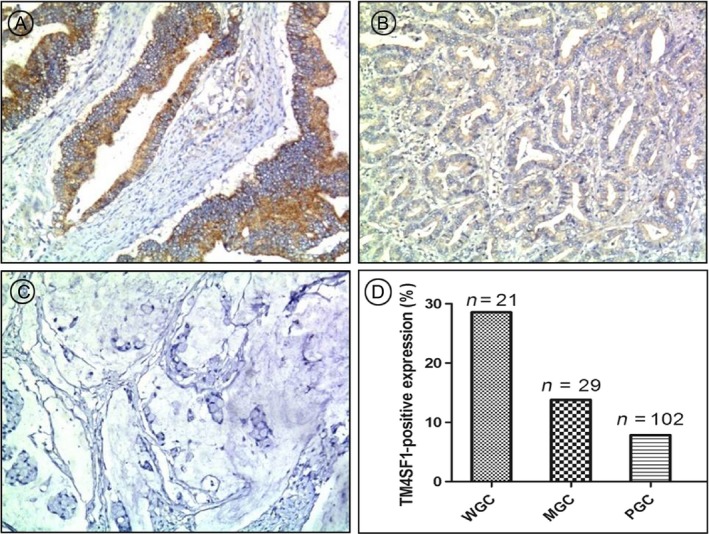
Higher levels and strong expression rate of TM4SF1 were associated with GC tissues of higher‐grade differentiation. TM4SF1 expression in GC tissues was determined by immunohistochemtry. Expression rates of TM4SF1 (D) in (A) well, (B) moderately, and (C) poorly differentiated GC tissues.

To investigate expression of TM4SF1 in GC tissues, we performed immunohistochemical staining using paraffin sections of GC tissue samples derived from 152 patients. Results showed that strong TM4SF1 staining was observed in well‐differentiated GC tissues (Fig. 2A), weak TM4SF1 staining in moderately differentiated GC tissues (Fig. 2B), and negative TM4SF1 staining in poorly differentiated GC tissues (Fig. 2C). Among 152 GC tissue samples, strong TM4SF1 staining was observed in 28.57% well‐differentiated GC tissue samples (6/21) (Fig. 2D), 13.79% moderately differentiated GC tissue samples (4/29) (Fig. 2D), and 7.84% poorly differentiated GC tissue samples (8/102) (Fig. 2D). These result suggested that higher levels and strong expression rate of TM4SF1 were associated with GC tissues of higher‐grade differentiation.

### TM4SF1 levels were lower in gastric cancer tissues than gastric noncanerous tissues

**Figure 3:**
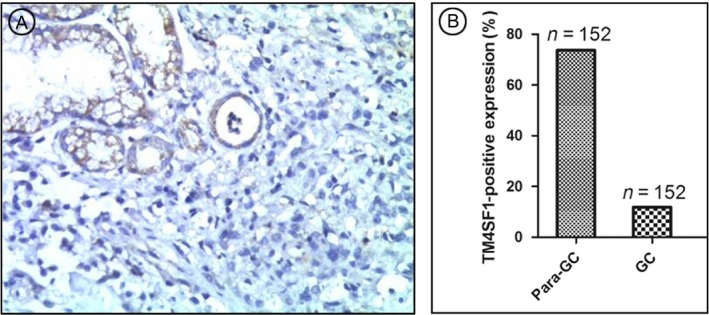
Transmembrane‐4‐L‐six‐family member‐1 expression rate was lower in GC tumor than para‐tumor tissues. TM4SF1 expression in GC tissues was determined by immunohistochemtry. (A) Expression of TM4SF1 in GC tissue samples and their adjacent noncancerous tissue samples. (B) Expression rates of TM4SF1 in GC tumor and para‐tumor tissue samples.

We examined the expression of TM4SF1 in GC tissue samples and their adjacent noncancerous tissue samples in the same resections undergone immunohistochemical analysis. The results showed that the negative expression of TM4SF1 in the GC tissues and positive expression of TM4SF1 in the adjacent noncancerous tissues in the same resections (Fig. 3A). In our patient cohort, immunohistochemical analysis showed that the rate of TM4SF1 expression was significantly lower in GC tissues (11.84%, 18/152) than that of noncancerous gastric mucosa tissues (73.68%, 112/152) (Fig. 3B).

**Figure 4:**
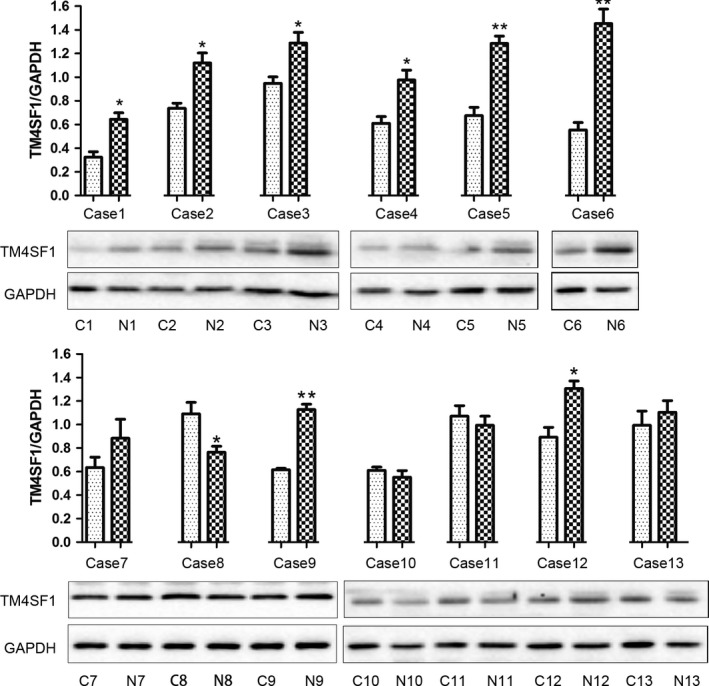
Transmembrane‐4‐L‐six‐family member‐1 levels were lower in gastric cancer tissues than gastric noncanerous tissues. TM4SF1 expression in GC tumor and para‐tumor tissue samples was determined by Western blotting. C, cancer tissue; N, normal tissue. GAPDH was a loading control and a normalization reference for quantitation.

To confirm that the TM4SF1 protein levels were lower in GC tissues than that of noncancerous gastric mucosa tissues, we compared TM4SF1 protein levels in 13 paired fresh‐frozen GC and the corresponding noncancerous gastric tissue samples by Western blotting. The results showed that these paired samples exhibited a lower TM4SF1 expression in the tumor tissues (61.54%, 8/13), compared with matched noncancerous tissue samples (7.69%, 1/13) (Fig. 4). These data suggested that TM4SF1 levels were lower in gastric cancer tissues than gastric noncanerous tissues.

### Association of TM4SF1 protein expression with the clinicopathologic factors of GC

We analyzed the association between TM4SF1 protein expression determined by immunohistochemistry and clinicopathological factors of 152 patients with GC using Chi‐square test. The results showed that the expression of TM4SF1 was negatively correlated with depth of invasion (*P *=* *0.031), nodal metastasis (*P *=* *0.042), TNM stage (*P *=* *0.030), and Lauren classification (*P *=* *0.026) (Table 1). There was no significant correlation between TM4SF1 expression and age, gender, tumor size, or distant metastasis (*P > *0.05, Table 1).

**Table 1:** Correlation between TM4SF1 protein expression and clinicopathologic features of GC

Clinicopathological features	All cases	TM4SF1 (%)		*P* value^a^
		Negative	Positive	
Age at diagnosis(years)				
<58	68	61 (89.71)	7 (10.29)	0.595
≥58	84	73 (86.90)	11 (13.10)	
Gender				
Male	101	90 (89.11)	11 (10.89)	0.610
Female	51	44 (86.27)	7 (13.73)	
Size(diameter)(cm)				
<4	102	88 (86.27)	14 (13.73)	0.305
≥4	50	46 (92.00)	4 (8.00)	
Depth of invasion				
T1	17	12 (70.59)	5 (29.41)	0.031
T2	25	20 (80.00)	5 (20.00)	
T3	92	85 (92.39)	7 (7.61)	
T4	18	17 (94.44)	1 (5.56)	
Nodal metastasis				
N0	52	42 (80.77)	10 (19.23)	0.042
N1/2	100	92 (92.00)	8 (8.00)	
Distant metastasis				
M0	134	116 (86.57)	18 (13.43)	0.098
M1	18	18 (100.00)	0 (0.00)	
TNM stage				
0	6	4 (66.67)	2 (33.33)	0.030
I	26	19 (73.08)	7 (26.92)	
II	43	40 (93.02)	3 (6.98)	
III	73	67 (91.78)	6 (8.22)	
IV	4	4 (100.00)	0 (0.00)	
Lauren classification				
Intestinal type	54	42 (77.78)	12 (22.22)	0.026
Mixed type	8	7 (87.50)	1 (12.50)	
Diffuse type	90	85 (94.44)	5 (5.56)	

a Chi‐Square test.

### Correlation between TM4SF1 expression and USP10, S100A12, Ki67, p53 expression in GC

We further examined the correlations between TM4SF1 expression and markers for cancer invasion and metastasis USP10, S100A12, p53, or Ki67 expression using Spearman's rank correlation analysis. The results showed that expression of TM4SF1 was not correlated with USP10 (*P *=* *0.157), S100A12 (*P *=* *0.479), p53 (*P *=* *0.249), or Ki67 (*P *=* *0.166) expression in GC (Table 2).

**Table 2:** Correlation among expression of TM4SF1, USP10, S100A12, P53, and Ki67

Protein	All cases	TM4SF1 expression		*r* value	*P* value^a^
		Negative	Positive		
USP10^b^					
Negative	34	32	2	0.145	0.157
Positive	63	53	10		
S100A12^c^					
Negative	47	43	4	0.071	0.479
Positive	54	47	7		
P53					
0	55	50	5	0.112	0.249
1+	16	14	2		
2+	11	10	1		
3+	26	21	5		
Ki67^d^					
0	14	11	3	0.409	0.166
1+	15	11	4		
2+	38	36	2		
3+	33	28	5		

a Spearman's rank correlation test.

b USP10 protein expression was not measured in eleven patients.

c S100A12 protein expression was not measured in seven patients.

d Ki67 protein expression was not measured in eight patients.

### Correlation between TM4SF1 expression and GC patients’ overall survival

**Figure 5:**
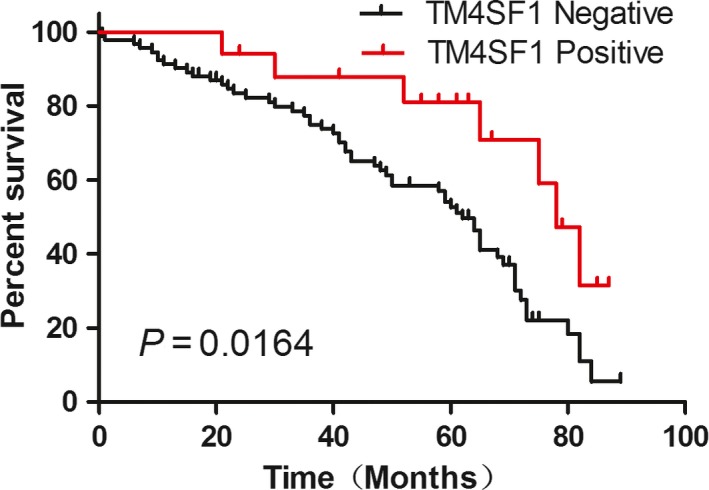
Expression of TM4SF1 was associated with well overall survival. Correlation between expression of TM4SF1 and overall survival was analyzed using Kaplan–Meier survival analysis and log‐rank test. Negative TM4SF1: score=0, *n* = 93; Positive TM4SF1: score≥1, *n* = 17. All statistical tests were two sides.

We evaluated the prognostic value of TM4SF1 on GC in 110 patients with postoperative follow‐up data using Kaplan–Meier analysis and the log‐rank test. The results showed that expression of TM4SF1 was associated with overall survival (*P *=* *0.0164) (Fig. 5). The 5‐year survival rate for patients with GC showing TM4SF1 positive was 58.82% (10/17), and the median survival time was 78 months, higher than that (12.90%, 12/93) of patients with GC showing TM4SF1 negative whose median survival time was 62 months.

## Discussion

In this study, we found that TM4SF1 protein levels were lower in gastric cancer tissues than gastric noncanerous tissues. Consistently, higher and strong TM4SF1 expression rate is associated with GC tissues of higher‐grade differentiation. These data suggested that low expression of TM4SF1 is associated with carcinogenesis and development of gastric cancer. This is different from lung, breast, colon, ovarian, renal, and prostate carcinomas where TM4SF1 mRNA and protein levels are highly expressed 19. Studies have shown that TM4SF1 is higher in pancreatic cancer tissues and cell lines than the control. Knockdown of TM4SF1 inhibited the migration and invasion of pancreatic cancer cells 13. Overexpression of TM4SF1 in HepG2 cells resulted in reduced apoptosis and increased cell migration in vitro and enhanced tumor growth and metastasis in vivo 20. It is likely that the role of TM4SF1 in carcinogenesis and development is tissue specific.

In the current study, we found that the expression of TM4SF1 was negatively correlated with depth of invasion, nodal metastasis, TNM stage, and Lauren classification, but not correlated with age, gender, tumor size, or distant metastasis. The results suggested that TM4SF1 was a tumor suppressor in GC progression and invasion. Similar result was also obtained in the study that showed contribution of TM4SF1 loss to the invasion and migration of pancreatic cancer cells 15. However, other study showed that high expression of TM4SF1 is correlated with T stage, TNM stage, lymph node metastasis status of MIBC (muscle invasive bladder cancer) patients 11. It seems that the role of TM4SF1 in cancer progression and invasion is also tissue‐specific.

We also evaluated the prognostic significance of TM4SF1 in patients with GC and found that lower expression of TM4SF1 was associated with poorer overall survival as revealed by Kaplan–Meier analysis, suggesting that TM4SF1 could be a novel prognostic marker for patients with GC. This is different from cancers such as muscle‐invasive bladder cancer 11, pancreatic Cancer 12, 13, and glioma 14 where overexpression of TM4SF1 is linked to poor prognosis of these cancers. Therefore, the role of TM4SF1 expression as a prognostic marker is dependent on the types of cancers.

USP10, S100A12, p53, and Ki67 are tumor molecular markers for cancer invasion and metastasis 21, 22, 23, 24. USP10 is a de‐ubiquitination enzyme, which removes ubiquitin from specific protein substrates, allowing protein salvage from proteasome degradation 21, 25. Lekishvili T et al. found that TM4SF1 was ubiquitylated and that ubiquitylation was essential for its function in cell migration 26. Our previous study indicated that S100A12 expression was correlated with the depth of invasion, and it was evaluated as an independent risk factor for poor overall survival of GC 22. p53 and Ki67 are established markers for cancer progression 27, 28. In the current study, we found that there was no correlation between expression of TM4SF1 and USP10, S100A12, p53, or Ki67 expression in GC, suggesting that TM4SF1 plays a distinct role in GC invasion and metastasis from those of USP10, S100A12, p53, or Ki67, although they all contribute to cancer invasion and metastasis. The exact role of TM4SF1 in GC invasion and metastasis deserves further investigation.

In the current study, we identified the gastric histological localization of TM4SF1 and found that TM4SF1 was heterogeneously expressed in normal gastric mucosa, with high levels in fundus mucosa. While TM4SF1 is a transmembrane protein 3, also found in the cytoplasm 29, and tentatively in nuclei of both cultured endothelial cells and tumor cells 9, and abundantly expressed on the plasma membrane and intracellular vesicles of lung, breast, colon, ovarian, renal, and prostate carcinomas and low expressed in normal vascular endothelium 7, 8, the cellular localization of TM4SF1 in gastric tissue remains obscure.

In conclusion, TM4SF1 is heterogeneously expressed in normal gastric mucosa, with a high expression rate in fundus mucosa. TM4SF1 protein levels were lower in gastric cancer tissues than gastric noncanerous tissues. Higher and strong TM4SF1 expression rate is associated with GC tissues of higher‐grade differentiation. Low expression of TM4SF1 is associated with carcinogenesis and development, tumor progression and invasion of gastric cancer, and poor overall survival of patients with GC. TM4SF1 is a tumor suppressor for GC and a novel prognostic marker for patients with GC, where the exact role of TM4SF1 remains and deserves further investigation.

## Conflict of Interest

No competing financial interest exists.
